# Search for Mutations Connected With Non‐Response to Anti‐EGFR Therapy in mCRC in the Morphologically Defined Regions of Primary Tumours

**DOI:** 10.1002/cam4.70910

**Published:** 2025-04-29

**Authors:** Martina Čarnogurská, Valeriia Serhiivna Vasylieva, Táňa Macháčková, Marie Boudná, Lucie Pifková, Jana Orlíčková, Tina Catela Ivkovic, Ondrej Slabý, Beatrix Bencsiková, Vlad Popovici, Eva Budinská

**Affiliations:** ^1^ RECETOX, Faculty of Science Masaryk University Brno Czech Republic; ^2^ Central European Institute of Technology, Masaryk University Brno Czech Republic; ^3^ Department of Biology, Faculty of Medicine Masaryk University Brno Czech Republic; ^4^ Masaryk Memorial Cancer Institute Brno Czech Republic

**Keywords:** anti‐EGFR therapy, *BRAF*, invasion front, *KRAS*, metastatic cancer, morphological sampling, *NRAS*, *PIK3CA*, PTEN, tumour heterogeneity

## Abstract

**Background:**

Emerging evidence suggests that tumour morphological heterogeneity may influence mutational profiles relevant to therapy response. In this pilot study, we aimed to assess whether mutations identified within specific morphological patterns or at the invasion front correlate with shorter time to progression after anti‐EGFR therapy, as compared to whole‐tissue analysis.

**Methods:**

We investigated genetic mutations in 142 samples from primary tumours of 39 *KRAS* wild‐type metastatic colorectal cancer (CRC) patients receiving anti‐EGFR therapy. Deep next‐generation sequencing was performed on whole‐tumour sections and six morphology‐defined tumour regions.

**Results:**

Mutations in genes linked to anti‐EGFR therapy response (*KRAS*, *BRAF*, *NRAS*, *PTEN* and *PI3KCA*) were found uniquely in the non‐responder group, with substantial variability across morphological sub‐regions. *BRAF* mutations were aligned with serrated and mucinous morphologies, while *KRAS* mutations (p.Lys147Glu and p.Ala146Thr) were associated with mucinous and desmoplastic morphologies. In all cases, the cumulative mutational profile from sub‐regions provided more details than that of the whole‐tumour profile.

**Conclusion:**

Our findings highlight that comprehensive analysis, considering morphological heterogeneity, is crucial for personalised CRC treatment strategies.

## 1 Introduction

Colorectal cancer is a major contributor to global cancer cases and mortality, ranking third in incidence but second in mortality [1, 2]. Metastatic colorectal cancer has a poor prognosis, with a 5‐year relative survival rate below 15%. While EGFR‐targeted therapy has improved survival for RAS wild‐type mCRC patients, its efficacy is limited, typically lasting only 8–10 months in responsive individuals [3, 4]. As therapeutic interventions continue, approximately 80% of initial responders develop resistance [5]. Identifying additional markers for treatment guidance and prognostic stratification remains crucial. The heterogeneity of tumours represents a challenge for targeted therapies. Baisse et al. found intratumoural heterogeneity (ITH) in 67% of advanced CRCs, highlighting the importance of examining gene alterations across multiple regions [6]. Subsequent research found that analysing a single tumour block resulted in inaccurate *KRAS* and *BRAF* status in 10%–30% of cases, requiring multi‐region analysis [7, 8, 9]. Büttner et al. [10] emphasised the need to include all tumour components for molecular assessment to detect drug resistance‐related alterations. Reggiani‐Bonetti et al. [11] supported this, linking CRC molecular ITH to tumour differentiation. Normanno et al. addressed the heterogeneity of the mutations predicting the resistance to anti‐EGFR treatment (*KRAS, NRAS, BRAF* and/or *PIK3CA*) in the primary CRC tumours in the context of the proportion of neoplastic cells [12]. Unfortunately, generally, tumour molecular profiling is performed from only one formalin‐fixed paraffin‐embedded (FFPE) block, ignoring the intrinsic ITH [13]. While FFPE allows selective material choice for molecular analysis, less common fresh frozen samples [14, 15] may undergo analysis without histopathological evaluation. Many studies omit a detailed tissue map during formalin fixation and paraffin embedding, leaving the heterogeneity among specific tumour locations largely unexplored. Consequently, established guidelines for assessing tissue sampling adequacy to accurately represent the molecular characteristics of the entire tumour are lacking. This presents a challenge, especially when treatment decisions before surgery rely on small biopsies from the tumour's surface, potentially leading to misguided decisions due to incomplete information. An alternative approach to identify all mutant subpopulations involves analysing multiple samples from various morphological tumour components. Our focus on the morphological basis for molecular phenotyping stems from previous observations correlating six morphological patterns with CRC molecular subtypes [16]. These morphological patterns were later confirmed to have specific molecular features, resulting in contradictory results of prognostic signatures [17]. Additionally, our analysis of four different FFPE blocks per tumour revealed that CRC tumours show high ITH with respect to these morphotypes, regardless of stage [18].

Here, in a pilot study, we aim to address the pivotal question of whether the previously described morphological patterns or the invasive front harbor specific mutations that could potentially be associated with shorter time‐to‐progression after the anti‐EGFR treatment (regardless of line of treatment), as compared to the analysis of whole sections. The morphological patterns by definition are defined by regions with low and high neoplastic cell content and can help guide the slide selection, enriching the samples in more homogeneous cells.

For this, we performed deep targeted sequencing of six different morphological regions and the invasion front of primary tumours from early‐ and late‐progressing patients. We compared the resulting profiles with those obtained from whole‐tumour sections, with the ultimate goal of refining the sampling process for better identification of patients more likely to experience early progression.

## 2 Methods

### 2.1 Sample Collection

Fully resected primary tumour FFPE samples from 39 KRAS wild‐type colon cancer patients treated with anti‐EGFR (cetuximab or panitumumab) + − chemotherapy (FOLFIRI or FOLFOX) were retrospectively selected from the Masaryk Memorial Cancer Institute's biobank. These included 19 early progressors (< 6 months post‐treatment) and 20 late progressors (> 12 months post‐treatment), regardless of the line of therapy, marked as EP and LP, respectively. The primary tumour samples spanned the years 2002–2020, while the anti‐EGFR treatments were administered between 2005 and 2020. The study assessed the effectiveness of anti‐EGFR therapy using median progression‐free survival (PFS). PFS was measured from the onset of treatment to disease progression or intolerable toxicity, confirmed through radiological imaging (CT or MRI) according to RECIST version 1.1 criteria. Time‐to‐progression after anti‐EGFR treatment was selected as a variable to define responders versus non‐responders, to minimise the influence of previous and subsequent treatment lines.

The study was approved by the Research Ethics Committee of Masaryk University (approval code EKV‐2018‐052, 5 April 2019). All patients provided written informed consent. The study adhered to the EU's ethical principles (2000/C364/01) and the Declaration of Helsinki (2013).

### 2.2 Biobanking Protocol

The biobanking protocol for FFPE slides adhered to ISO 23187 standards. The age of the FFPE blocks can affect DNA quality, potentially increasing mutational load in older blocks. In this dataset, no statistically significant difference was observed in FFPE block age between the compared groups (*p* > 0.05, *T*‐test). Additional mutation testing included BRAF (p.Val600Glu) and NRAS codons 12, 13 and 61, as per clinical guidelines.

### 2.3 Morphology Assessment, Sample Preparation and DNA Extraction

**FIGURE 1:**
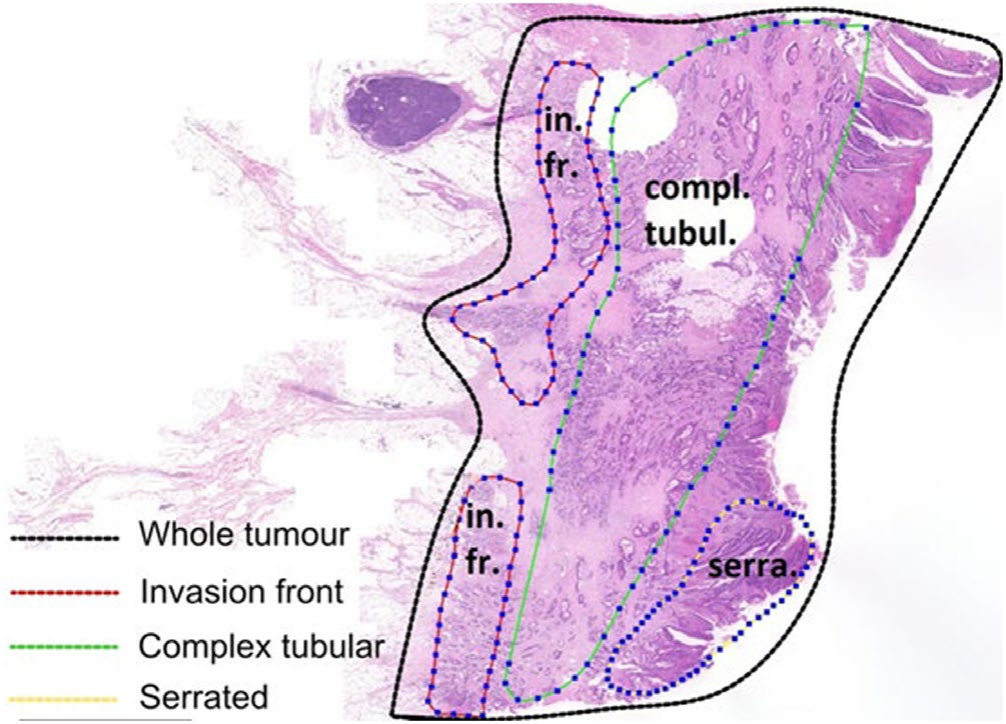
Schematic representation of the annotation of morphologically homogeneous regions on the FFPE slide.

**FIGURE 2:**
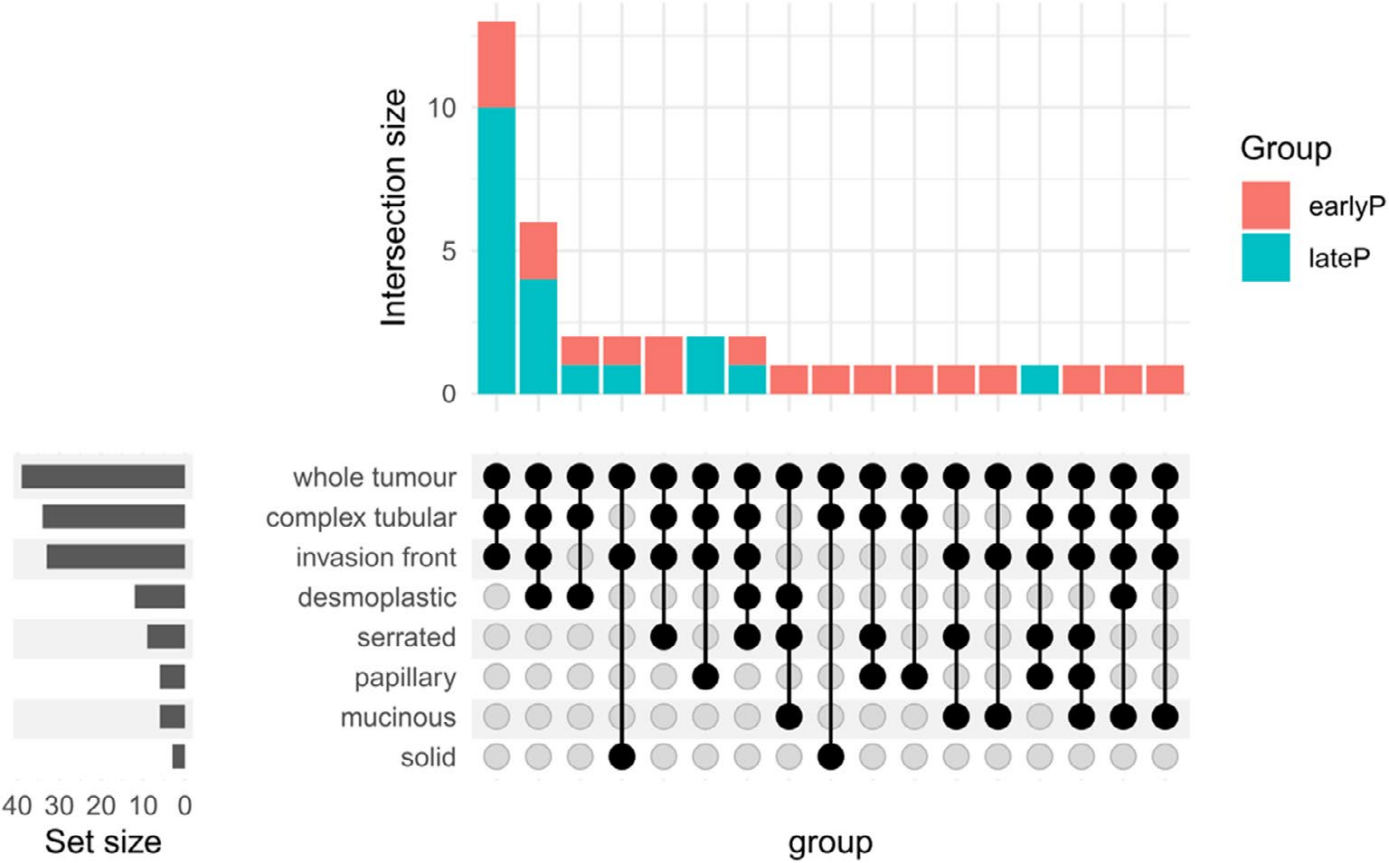
Distribution of the extracted regions across samples, stratified by group.

Tumour characteristics, determined by an expert pathologist per the 2019 WHO classification (5th ed.), included T‐stage, grade (G), localization, lymph node status and the presence of synchronous distant metastases. Patients with neoadjuvant treatment, multiple tumours or KRAS gene mutations in codons 12, 13, 61 and 146 were excluded. The FFPE blocks containing tumour tissue were then examined by an expert pathologist to identify morphological regions of interest. Blocks were sectioned in 3 μm serial sections. Morphological regions (as previously defined in [17]) were digitally marked in scanned whole slide images (at 20× magnification), see Figure 1. The invasion front was recognised in HE images as the spot where highly developed tumour cells infiltrate a stroma full of immune cells. A total of 142 samples (Table 1, Figure 2) were macrodissected, including regions of interest and whole‐tumour sections (macrodissected from the adjacent second section).

**TABLE 1:** Distribution of the extracted regions across groups.

Variable	Categories	Early progression (EP) (< 6 months)	Late progression (LP) (> 12 months)	*p*	Sum
Region type	Whole tumour	19	20	NA	39
	Invasion front	14	19	0.092	33
	Complex tubular	15	19	0.182	34
	Desmoplastic	6	6	1	12
	Mucinous	6	0	0.008	6
	Papillary	3	3	1	6
	Serrated	7	2	0.064	9
	Solid/trabecular	2	1	0.605	3
	All	72	70		142
Number of profiled regions per tumour	# Regions: # Tumours	3 regs: 8 4 regs: 8 5 regs: 2 6 regs: 1	3 regs: 12 4 regs: 6 5 regs: 2 6 regs: 0		

The DNA extraction was performed from FFPE histopathological slides using AllPrep DNA/RNA Kits (Qiagen) according to their specific manufacturer's instructions.

### 2.4 Library Preparation and Sequencing

Genomic DNA isolated from FFPE tissue was analysed by QIAseq DNA QuantiMIZE Assay Kit (Qiagen) to determine the quantity and quality of isolated samples. The sample input for library preparation was determined based on the DNA quality. According to the manufacturer's recommendation, 100 ng of DNA was used for high‐quality samples and 250 ng was used for low‐quality samples. Libraries were prepared using QIAseq Targeted Human Comprehensive Cancer Panel (Qiagen). In brief, genomic DNA samples were first fragmented, end‐repaired and A‐tailed. The prepared fragments were then ligated with an UMI‐containing (unique molecular identifier) adapter and sample index using QIAseq combinatorial dual indices 12‐Index I (for Illumina platform, Qiagen). Target enrichment was performed by PCR using a region‐specific primer and a universal primer complementary to the adapter, followed by a universal PCR for amplification of the libraries and addition of Illumina‐specific adapter sequences. Final libraries were quantified by a qPCR‐based method using QIAseq Library Quant Assay Kit (Qiagen). The libraries were pooled based on the manufacturer's recommendations and sequenced using NextSeq 500/550 Mid Output Kit v2.5 (300 Cycles, Illumina) with QIAseq custom sequencing primer for Read 1 (QIAseq A Read 1 Primer I) on NextSeq 500 NGS system (Illumina). Targeted sequencing was performed using QIAGEN Human Comprehensive Cancer QIASeq DNA Panel and 150 bp paired‐end reads at Illumina NextSeq 500.

### 2.5 Bioinformatics Processing, Variant Calling and Statistical Analysis

Quality of the raw fastq files was checked using fastqc (v. 0.11.9) and multiqc (v. 1.14). All the adapters (Nextera Transposase Adaptors sequence was taken from Trimmomatic collection) were trimmed (min length = 75 bp) and poor‐quality reads (min base quality = 25) were removed using cutadapt (v. 4.2). Mapping of the reads was performed by Burrows‐Wheeler Aligner (v. 0.7.17.) to the GRCH38.p14 human reference genome indexed by samtools (v. 1.11). UMI extraction and UMI‐based deduplication of the samples were done using UMI‐tools (v. 1.0.0).

For variant calling and annotation of samples formatted as BAM files, we employed the nf‐core/sarek pipeline (v.3.4.0). The variant calling was performed using Mutect2 (v.4.4.0). For annotation, we incorporated both SnpEff (v. 5.1d) and VEP (v.110), aligning these processes with the GRCh38 reference genome with the database for annotation ClinVar (v.202301).

To determine the coverage of the selected variants, we utilised BCFtools (v.1.14), ensuring precise and reliable coverage assessment. Only variants with FILTER values of PASS, germline, or panel_of_normals and annotated in the ClinVar database with a gene symbol assigned were considered in the analysis. This resulted in a dataset with 3126 variants. The coverage of positions of the 3126 variants was obtained also for samples without the variants called. If a coverage at the variant position was < 10, the information on the variant was considered “not available.” A variant was considered “pathogenic,” when it was annotated in the ClinVar database as pathogenic, likely pathogenic, or with conflicting interpretations of pathogenicity.

Differences in the distribution of continuous and categorical variables between the EP and LP groups were tested using the Wilcoxon rank sum test or Fisher's exact test, respectively. The differences in age were tested using a two‐sided two‐sample *T*‐test. We considered results significant at *α* < 0.05. All statistical analyses were performed in R 4.2.1 [19].

## 3 Results

We analysed 142 samples from 39 KRAS wild‐type fully resected primary tumours of metastatic CRC patients undergoing anti‐EGFR therapy (in first‐, second‐ or third‐line treatment), covering the entire tumour, invasion front and six morphological regions. 75% and 80% of patients in the LP and EP group (respectively) presented at diagnosis as stage IV.

The whole‐tumour section was profiled in all tumours. Invasion front was profiled in 14 and 19 tumours of the EP and LP groups, respectively. The number of profiled regions per tumour ranged from 3 to 6 (based on availability), with most tumours having 3 or 4 regions (20 and 14 cases, respectively) (Table 1). Some combinations of morphologies were not observed, such as TB with MU, DE, SE or PP and DE with PP (Figure 2).

All tumours were classified as adenocarcinoma except for one case of mucinous adenocarcinoma and one case of signet‐ring cell carcinoma (both in EP group). Interestingly, all six tumours with mucinous regions belonged to the EP group, and this group was also enriched in serrated morphologies (7 compared to 2) (Table 1).

We did not reject the hypothesis of similar distribution between the two compared groups for all clinical variables but site and the line of anti‐EGFR treatment (Table 2). 40% of tumours from the EP group were from the right side of the colon, while 80% of right‐sided tumours belonged to the EP group. At the same time, the EP group underwent anti‐EGFR therapy more often in the second or third line treatment.

**TABLE 2:** Distribution of clinical variables at diagnosis of primary tumour and number of detected variants between the early‐ and late‐progressing patients.

	Early progression (< 6 months)	Late progression (> 12 months)	*p*	Test
*N*	19	20		
Gender = M (%)	11 (57.9)	15 (75.0)	0.320	Fisher's exact test
Age (mean (SD))	59.16 (7.97)	60.45 (10.99)	0.678	Student's *T*‐test
Grade (%)				
1	3 (15.8)	2 (10.0)	0.731	Fisher's exact test
2	12 (63.2)	16 (80.0)		
3	3 (15.8)	1 (5.0)		
9	1 (5.3)	1 (5.0)		
T‐stage (%)				
T2	2 (10.5)	0 (0.0)	0.483	Fisher's exact test
T3	13 (68.4)	13 (65.0)		
T4	4 (21.1)	6 (30.0)		
Tx	0 (0.0)	1 (5.0)		
N‐stage (%)				
N0	3 (15.8)	5 (25.0)	0.065	Fisher's exact test
N1	6 (31.6)	11 (55.0)		
N2	10 (52.6)	3 (15.0)		
Nx	0 (0.0)	1 (5.0)		
Synchronous metastases = M1 (%)	16 (84.2)	15 (75.0)	1.000	Fisher's exact test
AJCC stage (%)				
II	2 (10.5)	1 (5.0)	0.492	Fisher's exact test
III	1 (5.3)	4 (20.0)		
IV	16 (84.2)	15 (75.0)		
Site				
Right	7 (36.8)	2 (10.0)	0.039	Fisher's exact test
Transverse	0 (0.0)	3 (15.0)		
Left	11 (57.9)	11 (55.0)		
Rectosigmoid	0 (0.0)	3 (15.0)		
Rectum	1 (5.3)	1 (5.0)		
Histology				
Adenocarcinoma	17	20	0.231	Fisher's exact test
Mucinous adenocarcinoma	1	0		
Signet‐ring cell carcinoma	1	0		
Number of unique variants per tumour, median (IQR)	297 (109.5)	242.5 (58.5)	0.028	Wilcoxon rank sum test
Number of unique pathogenic variants per tumour, median (IQR)	17 (12.5)	5 (6.75)	0.002	Wilcoxon rank sum test

There were 395 pathogenic variants, distributed across 119 genes. Of these, the most frequently mutated (in terms of number of patients with more than 10 pathogenic variants in that gene) were *HNF1A* (31), *BRCA2* (15), *TP53* (12), *PTCH1* (11), *BRCA1* (11) and *PDGFRA* (10).

The number of detected variants per region ranged between 48 and 231 (median 152), while the number of detected pathogenic variants per region ranged between 0 and 18 (median 4). The number of detected variants per primary tumour (regardless of the type of region sampled) ranged between 155 and 465 (median 252), and the number of detected pathogenic variants per tumour ranged between 1 and 39 (median 10). The genes with the largest number (≥ 10) of unique variants found across all samples were *BRCA2* (39), *TSC2* (14), *TP53* (13), *PTCH1* (14), *BRCA1* (13), *NF1* (11), *MLH1* (11), *APC* (11) and *KMT2D* (11).

### 3.1 Variants Between Early and Late Progression Group

With respect to response to therapy, we first examined unique pathogenic variants per tumour (regardless of the region of origin). Tumours from the EP group showed higher mutational burden compared to the LP group either when assessing all variants (median 297 for EP vs. 242.5 for LP, *p* = 0.028) or pathogenic variants (median 17 vs. 5, *p* = 0.002) (Table 2).

**FIGURE 3:**
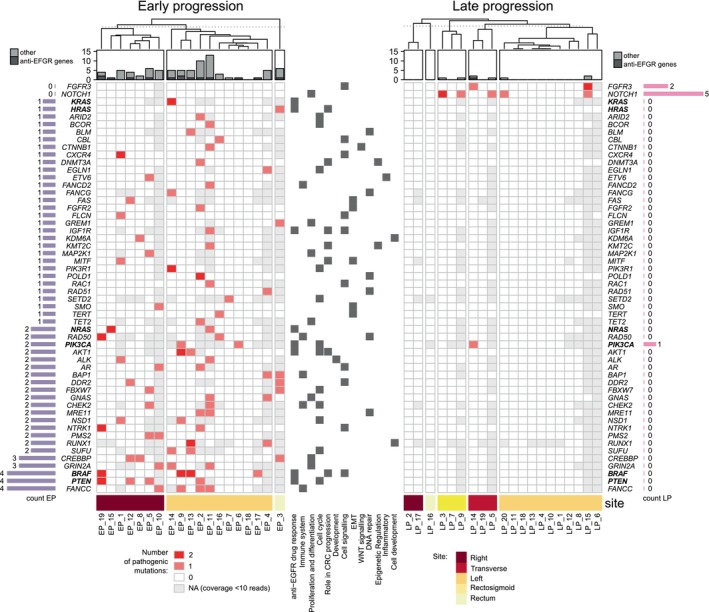
Presence of any pathogenic mutations in genes known for their role in anti‐EGFR therapy response and in other genes specific for the early‐ or late‐progressing group.

We then focused on genes with at least one pathogenic variant specific to the EP or LP group (Figure 3). Again, tumours from the EP group harboured more specific genes with pathogenic variants than tumours from the LP group (54 vs. 2). Only one tumour from the EP group showed none of the EP‐specific pathogenic variants, while as much as 70% (14/20) tumours in the LP group showed none of the LP‐specific pathogenic variants.

The most frequently mutated specific genes in the EP group were *BRAF*, *PTEN* and *FANCC* and *NOTCH1* in the LP group.

In the EP group, 12 tumours had pathogenic mutations in key genes associated with response to anti‐EGFR therapy (1 × in *BRAF* + *PTEN*, 1 × in *BRAF* + *PIK3CA*, 2 × in *BRAF*, 3 × in *PTEN*, 2 × in *NRAS*, 1 × in *KRAS*, 1 × in *HRAS* and 1 × in *PIK3CA*). *BRAF* p.Val600Glu mutations were found in three left‐sided and 1 right‐sided tumour, *NRAS* p.Gln61Lys in 1 right and 1 left‐sided tumour, *KRAS* p.Lys147Glu and p.Ala146Thr in one left‐sided tumour, and *HRAS* p.Ala121Thr in the tumour of the rectum. *PTEN* p.Asp162Glu, p.Phe21fs, and *PTEN* 1027‐1G > T mutations were found in three right‐sided and one left‐sided tumour, and *PIK3CA* p.Gln546Lys and p.Gln546Glu mutations in two left‐sided tumours.

Other mutations specific to the EP group were associated with crucial biological processes as shown in Figure 3.

### 3.2 Morphology‐Specific Mutations and Within‐Tumour Case Studies

The within‐tumour profile similarity, computed on a full set of 3126 variants (Table S1) and based on Manhattan distance, was higher than the between‐tumour similarity (average similarity 0.63 vs. 0.41). However, when restricting the similarity to pathogenic mutations only, the between‐tumour similarity was almost equal to the within‐tumour (average 0.74 vs. 0.81) and, for 10 cases, was even higher.

We then restricted the analysis to the distribution of pathogenic mutations within the tumour regions (Figure S1). We did not seek pathogenic mutations that would be specific to a certain type of morphology (the design of the experiment does not allow for such an exercise), but we did observe distinctive traits in the tumour fractions. We detected both previously reported (*HNF1*, *SUFU*) and unreported (*BRCA2*, *GRIN2A*) alterations associated with mucinous adenocarcinoma. Further, at the invasion front, we observed mutations in genes such as *RAD50*, *SMAD4*, *MSH2* and *PTCH1* which may contribute to increased invasiveness, metastasis, and treatment resistance, highlighting the dynamic nature of the invasion front. Other mutations, such as *BRAF*, were most often detected in all sampled regions of the tumour.

**FIGURE 4:**
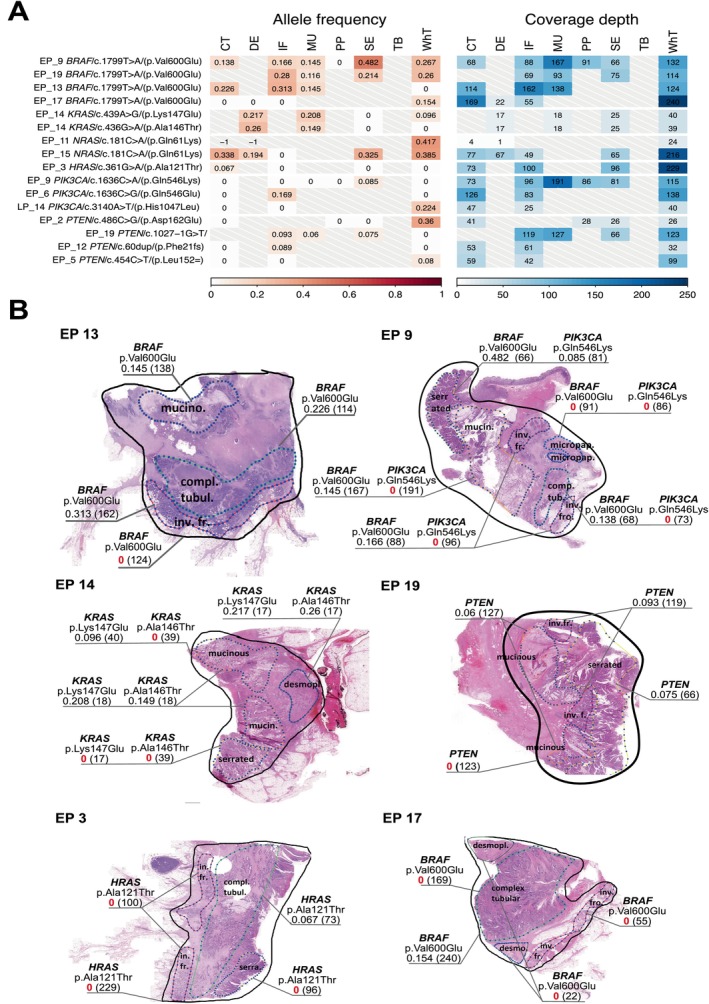
Variability of selected mutations across the morphological regions. (A) All pathogenic mutations of selected genes involved in response to anti‐EGFR therapy and tumours in which they were found (in any of the regions). Heatmaps show allele frequencies (left) and coverage depths (right) for each variant. Each row represents a patient and the position at which the variant occurred. (B) Annotated FFPE scans of selected tumours with allele frequencies and coverage depths of selected pathogenic mutations in genes implicated in anti‐EGFR therapy response.

Interestingly, the whole‐tumour sections never comprised complete mutational profiles as found in tumour's subsampled regions, especially those characterised by lower cell counts, such as mucinous or desmoplastic. Also, the whole‐tumour sample often inadequately reflected the molecular profile of the invasion front. Inspection of variants in genes linked to anti‐EGFR therapy (*KRAS*, *BRAF*, *NRAS*, *PTEN* and *PIK3CA*) revealed variability across tumours and fractions (Figure 4A). *PIK3CA* was considered alongside *PTEN* due to their uncertain associations with anti‐EGFR therapy response. *BRAF* mutations correlated with serrated and mucinous morphologies, *KRAS* mutations with mucinous and desmoplastic morphologies. Below, we depict six cases showing variant differences between morphological sites. Patient EP13 exemplifies this complexity as the *BRAF* p.Val600Glu was tested negative in the whole‐tumour sample but positive in mucinous, complex tubular and invasion front regions. This patient also tested *BRAF* p.Val600Glu positive in the clinical testing. In contrast, patient EP17, *BRAF* p.Val600Glu negative in clinical testing, exhibited positivity solely in the whole‐tumour section, including both the selected regions and interstitial regions not associated with a specific morphological type. The occurrence of Ras mutations (*KRAS* and *HRAS*) exhibited variability across distinct morphological regions as well. Specifically, in patient EP14, while we did not find any of the clinically tested *KRAS* mutations, we observed two additional *KRAS* mutations: *KRAS* p.Lys147Glu and *KRAS* p.ALA146THR. The *KRAS* p.Lys147Glu was negative in the serrated region, and *KRAS* p.ALA146THR was negative both in the serrated and the whole‐tumour section. In patient EP3, *HRAS* p.Ala121Thr was exclusively detected in the complex tubular region, but not in the serrated fraction, nor on the invasion front, nor in the whole tumour. Patient EP9 (in clinical testing positive for *BRAF* p.Val600Glu) exhibited disparities in the detection of *BRAF* p.Val600Glu and *PIK3CA* p.Gln546Lys mutations. Notably, differences in this patient were observed in the serrated region, which exclusively tested positive for the *PIK3CA* p.Gln546Lys mutation among all other regions, and the micropapillary region, which conversely tested negative for the *BRAF* p.Val600Glu mutation. Also, the detection of *PTEN* 1027‐1G > T in patient EP19 was inconclusive, while it tested positive in the dissections of morphological regions but negative for the dissected whole tumour.

In patients EP9 and EP19, who both tested positive for *BRAF* p.Val600Glu in clinical testing, we found this mutation in all the regions. Similarly, patient EP15 (not tested for *NRAS* in the clinical testing) showed *NRAS* p.Gln61Lys mutation in all the regions. Another patient EP11, which was also not tested for *NRAS* in the clinical testing, tested positive for *NRAS* p.Gln61Lys in the whole tumour only, due to lack of coverage of this variant in other regions.

## 4 Discussion

Despite the substantial impact of anti‐EGFR monoclonal antibodies (anti‐EGFR mAbs) combined with chemotherapy on mCRC, their therapeutic efficacy is constrained by the development of drug resistance. Moreover, the duration of clinical benefit among responders to anti‐EGFR mAbs therapy is limited [3, 4]. In our study and motivated by our previous research [17], we linked histological morphology and molecular pathology in anti‐EGFR treatment by analysing mutational profiles of tumour regions (consisting of selected morphotypes, invasion front and whole‐tumour sections) of the early‐ and late‐progressing patients.

The analysis of unique pathogenic variants per tumour, irrespective of their region of origin, revealed higher mutational burden in the EP group compared to the LP group, both with all variants and only the pathogenic ones. This may reflect the underlying genetic complexity and aggressive nature of these tumours, contributing to their early progression [20]. The within‐tumoural similarity of pathogenic mutational profiles was less prominent than that of full mutational profiles, indicating that pathogenic mutations represented known oncogenic processes with added tumour region‐specificity. Most importantly, we observed that the whole tumour's mutational profile did not represent the sum of constituent regions.

Mutations in the EP group revealed complex genetic interplay in cancer development. Notably, 12/19 EP patients had pathogenic mutations in one or several genes linked to poor anti‐EGFR therapy response: *KRAS, BRAF, NRAS, HRAS, PTEN* and *PIK3CA*. The most frequently mutated genes were *BRAF, PTEN* and *FANCC*.

Several mutations of *KRAS* and other *RAS* genes were already linked to poor response to anti‐EGFR treatment [21, 22, 23, 24, 25, 26, 27, 28]. While mutations in the *KRAS* gene are frequent (~45% of CRC), the tested regions for treatment selection are limited to exon 2 at codons 12 and 13. Similarly, *NRAS* mutations (~1% of CRC [29]) tested cover the codons 12, 13 and 61. Still, oncogenic mutations outside these commonly tested loci have been identified [30, 31, 32] and we also found this to be the case in one patient who harboured two other variants (p.Lys147Glu, p.Ala146Thr). Mutations of the *BRAF* gene are a negative predictor for anti‐EGFR treatment response and indicators of poor prognosis in metastatic colorectal cancer (mCRC). They affect 8%–12% of cases, with V600 amino acid alterations being the most common [21, 33]. We identified this mutation in 4/19 EP patients, with one testing positive despite negative previous clinical tests. Previous research has demonstrated that analysing DNA from a single block can erroneously classify the *KRAS* and *BRAF* status as wild type in up to 30% of patients [7, 9, 34]. *PIK3CA* mutations and loss of *PTEN* were proposed as prognostic or predictive biomarkers for anti‐EGFR mAb in mCRC patients with wild‐type RAS [35, 36, 37]. We identified mutations in these genes in six patients triple‐negative for *NRAS, KRAS* and *HRAS*. In two patients, *PTEN* or *PIK3CA* mutations coincided with a *BRAF* mutation. Notably, positivity of these mutations varied significantly across morphological regions in all six cases. Our findings on RAS/RAF mutations reveal disparities across tumour regions, with heterogeneous distribution observed in certain studies [38, 39, 40] but inconsistently replicated in others [41, 42]. This significant heterogeneity within tumours regarding specific mutations could impact therapeutic strategies, as tumours with molecular alterations in only a subset of cells may show reduced sensitivity to targeted therapies, as seen in our findings, where patients tested negative overall but positive in specific tumour regions or vice versa.

*BRAF* mutations were associated with serrated and mucinous morphologies. In contrast, *KRAS* mutations, specifically the variants p.Lys147Glu and p.Ala146Thr, which are not typically included in standard clinical testing panels, were associated with mucinous and desmoplastic morphologies.

Our findings extend the current literature by demonstrating that not only the selection of an inappropriate tumour block but also the ITH within each block can compromise the accurate identification of RAS/RAF mutations [7, 8, 9]. *PIK3CA* exon 20 mutations may reduce anti‐EGFR mAb responsiveness, but clinical applicability is uncertain due to small sample sizes and potential confounding factors. Similarly, *PTEN* status association with treatment response remains inconclusive due to challenges in assessment.

The co‐occurrence of *PIK3CA* and *PTEN* alterations with *KRAS* or *BRAF* mutations further complicates their evaluation [21, 43, 44, 45]. The role of *PIK3CA* mutation and *PTEN* status in mediating EGFR‐directed therapy resistance in colorectal cancer is still unclear [21, 43, 46, 47, 48]. The detection of mutations in PTEN and PIK3CA appeared to be very sensitive to the sampled regions, suggesting not only the need for a more detailed sampling, but also raising the question of whether their prevalence is underestimated.

Our analysis revealed that 40% of EP group tumours were right‐sided, and, reciprocally, eight out of 10 right‐sided tumours were in the EP group. It was shown that right‐sided tumours exhibit lower responsiveness to anti‐EGFR treatment [49] and higher mutation rates in genes like *BRAF, PIK3CA*, and *KRAS* [50], suggesting potential underlying reasons for this association. In our data, these also comprised 3 EP tumours without any of the mutations involved in the resistance to the anti‐EGFR treatment. The mutations of the remaining four EP patients were not linked to anti‐EGFR therapy resistance until now, suggesting that early progression could be influenced by rare events beyond the detection power of relatively small sample size sets or detection thresholds used in most studies.

In the LP group, specific genes were found in only 6/20 patients: FGFR3 (2/20) and NOTCH1 (5/20). This might be due to Notch signalling's role in tumour recurrence and progression [51, 52]. However, our attempts to define up to 14 patients as belonging to the LP group based on mutation similarity proved unsuccessful. While EP mutations suggest faster progression, in the LP group, it appears that correct treatment was initiated, indicating that other factors likely contributed to the later progression.

Our findings show that when sampling the tumour for molecular pathology analysis, it is prudent to take into account the anticipated presence or absence of mutations in the context of intratumoral morphological heterogeneity. This has implications in both research and clinical practice. Research‐wise, the identification of additional relevant mutations in non‐responders to anti‐EGFR therapy might be possible only when focused on morphological regions, as exemplified by our finding of *KRAS* (p.Lys147Glu and p.Ala146Thr) mutations in mucinous and desmoplastic, but not serrated regions of a tumour, despite the latter having a larger proportion of neoplastic cells. Additionally, our findings indicate that mutation frequencies, such as those of *PIK3CA* and *PTEN*, may be underestimated due to the non‐specificity of the regions examined (in our case these were present in very specific regions of the tumour such as IF, etc.). From a clinical perspective, for instance, we observed that *BRAF* mutations occurred in tumours with serrated morphology. Hence, in the case of a negative *BRAF* result when using a classical (and widely heterogenous) sampling approach, the presence of serrated morphology could indicate focal sample acquisition from this region for further testing. These insights should be carefully considered when procuring samples. These observations are even more important with respect to our previous results (https://www.biorxiv.org/content/10.1101/2024.04.10.588907v1.full), that clearly show that serrated and desmoplastic regions rarely co‐occur and that there is a statistically significant increase in desmoplastic region proportion in the primary tumours of stage IV CRC.

Looking ahead, ctDNA has the potential to enhance diagnostic precision by capturing tumour heterogeneity in cases where tissue biopsies are insufficient or unavailable. However, its current limitations, including lower sensitivity compared to tissue‐based NGS, emphasise the need for technological advancements and validation to integrate ctDNA effectively into clinical practice [53, 54].

Indeed, intratumoral heterogeneity complicates biomarker detection and treatment decisions. Studies [55, 56] show that genetic subclonal dynamics play a key role in tumour evolution and resistance to therapy, which suggests that multi‐regional sequencing combined with AI‐assisted histopathology is the future in precision diagnostics. Moreover, combining ctDNA analysis with spatially resolved tumour profiling may even further improve biomarker detection and optimise targeted treatments.

Our preliminary investigation has some limitations. The study was retrospective with a restricted sample size, possibly introducing selection bias or confounding variables. Specifically, the variation in treatment lines and concurrent chemotherapy could have influenced the observed differences, emphasising the exploratory nature of these findings. Larger cohorts are needed to estimate the prevalence of cases affected by this variability. Still, our results clearly expose the challenges in accurately characterising the mutational landscape of the tumours, with a direct impact on treatment decisions. Whether morphological assessment by a pathologist or other means (such as AI based image analysis) is used as a basis for tumour sampling remains an open question, but the message is that multiple sites per tumour need to be interrogated to better capture its features. Furthermore, we acknowledge that validation in an independent dataset would significantly enhance the robustness of our findings. Currently, access to an equivalent dataset with similar morphology‐resolved molecular data is unavailable, limiting our ability to perform external validation. Future studies should focus on larger and more diverse patient cohorts or develop collaborative data‐sharing initiatives to validate and extend our findings, ultimately improving their translational relevance.

## 5 Conclusions

Overall, our study supports the notion that morphological patterns can guide minimal tumour sampling coverage to identify relevant mutations relevant for prediction of response to anti‐EGFR therapy. This prompts a reassessment of sampling practices and provides insights for diagnostics and profiling strategies. The selected morphological patterns are relatively easy to recognise by the pathologists (or an AI‐trained model), have been shown to be connected with molecular characteristics of the tumours and hence they can provide a guidance for a minimal tumour sampling coverage to identify relevant mutations. This approach can improve the accuracy of mutation detection and potentially provide better prognostic information, ultimately informing treatment strategies for metastatic colorectal cancer (mCRC) patients.

## Author Contributions

**Martina Čarnogurská:** conceptualization (equal), data curation (equal), investigation (equal), methodology (equal), visualization (equal), writing – original draft (lead), writing – review and editing (equal). **Valeriia Serhiivna Vasylieva:** formal analysis (equal), writing – original draft (equal). **Táňa Macháčková:** methodology (equal), writing – original draft (equal). **Marie Boudná:** methodology (equal), writing – original draft (equal). **Lucie Pifková:** methodology (equal), writing – original draft (equal). **Jana Orlíčková:** methodology (equal), writing – original draft (equal). **Tina Catela Ivkovic:** methodology (equal), writing – original draft (equal). **Ondrej Slabý:** conceptualization (equal), methodology (equal), project administration (equal), supervision (equal), writing – original draft (equal). **Beatrix Bencsiková:** conceptualization (equal), project administration (equal), resources (equal), writing – original draft (equal). **Vlad Popovici:** conceptualization (lead), data curation (equal), formal analysis (equal), investigation (equal), methodology (equal), project administration (equal), resources (equal), supervision (equal), writing – original draft (equal). **Eva Budinská:** conceptualization (equal), data curation (equal), investigation (equal), project administration (equal), resources (equal), supervision (equal), visualization (equal), writing – original draft (lead).

## Conflicts of Interest

The authors declare no conflicts of interest.

## Supporting information


**Figure S1.** Distribution of pathogenic variants in whole tumour, invasion front and across the morphological regions. Each row represents sample, each column represent mutations. Higlighted (bold) are mutations associated with non‐response to anti‐EGFR therapy.

File S1.

**Table S1.** List of all 3126 variants selected for analysis, annotated by ClinVar with available GeneSymbol.

## Data Availability

Full raw data collection (demultiplexed fastq files) and corresponding variant calling results (VCF) were uploaded to Zenodo (see File S1 for links to datasets).

## References

[1] H. Sung , J. Ferlay , R. L. Siegel , et al., “Global Cancer Statistics 2020: GLOBOCAN Estimates of Incidence and Mortality Worldwide for 36 Cancers in 185 Countries,” CA: A Cancer Journal for Clinicians 71, no. 3 (2021): 209–249. 33538338 10.3322/caac.21660

[2] R. L. Siegel , K. D. Miller , H. E. Fuchs , and A. Jemal , “Cancer Statistics, 2022,” CA: A Cancer Journal for Clinicians 72, no. 1 (2022): 7–33. 35020204 10.3322/caac.21708

[3] J. Tabernero , E. Van Cutsem , E. Díaz‐Rubio , et al., “Phase II Trial of Cetuximab in Combination With Fluorouracil, Leucovorin, and Oxaliplatin in the First‐Line Treatment of Metastatic Colorectal Cancer,” Journal of Clinical Oncology 25, no. 33 (2007): 5225–5232. 18024868 10.1200/JCO.2007.13.2183

[4] M. Borner , D. Koeberle , R. Von Moos , et al., “Adding Cetuximab to Capecitabine Plus Oxaliplatin (XELOX) in First‐Line Treatment of Metastatic Colorectal Cancer: A Randomized Phase II Trial of the Swiss Group for Clinical Cancer Research SAKK,” Annals of Oncology 19, no. 7 (2008): 1288–1292. 18349029 10.1093/annonc/mdn058

[5] A. Bardelli and S. Siena , “Molecular Mechanisms of Resistance to Cetuximab and Panitumumab in Colorectal Cancer,” Journal of Clinical Oncology 28, no. 7 (2010): 1254–1261. 20100961 10.1200/JCO.2009.24.6116

[6] B. Baisse , H. Bouzourene , E. P. Saraga , F. T. Bosman , and J. Benhattar , “Intratumor Genetic Heterogeneity in Advanced Human Colorectal Adenocarcinoma,” International Journal of Cancer 93, no. 3 (2001): 346–352, 10.1002/ijc.1343. 11433398

[7] L. Farber , E. Efrati , H. Elkin , et al., “Molecular Morphometric Analysis Shows Relative Intra‐Tumoural Homogeneity for KRAS Mutations in Colorectal Cancer,” Virchows Archiv 459, no. 5 (2011): 487–493. 22016105 10.1007/s00428-011-1158-y

[8] M. Jeantet , D. Tougeron , G. Tachon , et al., “High Intra‐ and Inter‐Tumoral Heterogeneity of RAS Mutations in Colorectal Cancer,” International Journal of Molecular Sciences 17, no. 12 (2016): 2015. 27916952 10.3390/ijms17122015 PMC5187815

[9] H. G. Jones , G. Jenkins , N. Williams , et al., “Genetic and Epigenetic Intra‐Tumour Heterogeneity in Colorectal Cancer,” World Journal of Surgery 41, no. 5 (2017): 1375–1383. 28097409 10.1007/s00268-016-3860-z PMC5394146

[10] J. Büttner , K. Jöhrens , F. Klauschen , et al., “Intratumoral Morphological Heterogeneity Can Be an Indicator of Genetic Heterogeneity in Colorectal Cancer,” Experimental and Molecular Pathology 104, no. 1 (2018): 76–81. 29337243 10.1016/j.yexmp.2018.01.007

[11] L. Reggiani‐Bonetti , V. Barresi , S. Bettelli , C. Caprera , S. Manfredini , and A. Maiorana , “Analysis of KRAS, NRAS, PIK3CA, and BRAF Mutational Profile in Poorly Differentiated Clusters of KRAS‐Mutated Colon Cancer,” Human Pathology 62 (2017): 91–98. 28025078 10.1016/j.humpath.2016.12.011

[12] N. Normanno , A. M. Rachiglio , M. Lambiase , et al., “Heterogeneity of KRAS, NRAS, BRAF and PIK3CA Mutations in Metastatic Colorectal Cancer and Potential Effects on Therapy in the CAPRI GOIM Trial,” Annals of Oncology 26, no. 8 (2015): 1710–1714. 25851630 10.1093/annonc/mdv176

[13] M. Fassan , “Molecular Diagnostics in Pathology: Time for a Next‐Generation Pathologist?,” Archives of Pathology & Laboratory Medicine 142, no. 3 (2018): 313–320. 29494219 10.5858/arpa.2017-0269-RA

[14] A. Lamont and R. Pottie , “Versatility of Soft‐Tissue Free Tissue Transfers,” South African Journal of Surgery 26, no. 3 (1988): 98–101. 3187794

[15] S.‐W. Pang , N. J. Awi , S. Armon , et al., “Current Update of Laboratory Molecular Diagnostics Advancement in Management of Colorectal Cancer (CRC),” Diagnostics (Basel) 10, no. 1 (2019): 9, 10.3390/diagnostics10010009. 31877940 PMC7168209

[16] E. Budinska , V. Popovici , S. Tejpar , et al., “Gene Expression Patterns Unveil a New Level of Molecular Heterogeneity in Colorectal Cancer,” Journal of Pathology 231, no. 1 (2013): 63–76. 23836465 10.1002/path.4212 PMC3840702

[17] E. Budinská , M. Hrivňáková , T. C. Ivkovic , et al., “Molecular Portraits of Colorectal Cancer Morphological Regions,” eLife 12 (2023): RP86655. 37956043 10.7554/eLife.86655 PMC10642970

[18] M. P. Dragomir , V. Popovici , S. Schallenberg , et al., “A quantitative tumor‐wide analysis of morphological heterogeneity of colorectal adenocarcinoma,” 2024 bioRxiv 2024.04.10.588907, 10.1101/2024.04.10.588907. PMC12163513 40511583

[19] R Core Team , R: A Language and Environment for Statistical Computing (R Foundation for Statistical Computing) (2022).

[20] J. Peng , Y. Liu , W. Li , et al., “Application of Tumor Burden Score for Predicting Conversion Outcome in Patients With Initially Unresectable Colorectal Liver Metastases After First‐Line Systemic Therapy,” Therapeutic Advances in Gastroenterology 14 (2021): 1–16. 10.1177/17562848211066206 PMC8721375 34987612

[21] P. Laurent‐Puig , A. Cayre , G. Manceau , et al., “Analysis of PTEN, BRAF, and EGFR Status in Determining Benefit From Cetuximab Therapy in Wild‐Type KRAS Metastatic Colon Cancer,” Journal of Clinical Oncology 27, no. 35 (2009): 5924–5930. 19884556 10.1200/JCO.2008.21.6796

[22] J.‐Y. Douillard , K. S. Oliner , S. Siena , et al., “Panitumumab‐FOLFOX4 Treatment and RAS Mutations in Colorectal Cancer,” New England Journal of Medicine 369, no. 11 (2013): 1023–1034. 24024839 10.1056/NEJMoa1305275

[23] C. Cremolini , D. Rossini , E. Dell'Aquila , et al., “Rechallenge for Patients With RAS and BRAF Wild‐Type Metastatic Colorectal Cancer With Acquired Resistance to First‐Line Cetuximab and Irinotecan: A Phase 2 Single‐Arm Clinical Trial,” JAMA Oncology 5, no. 3 (2019): 343–350, 10.1001/jamaoncol.2018.5080. 30476968 PMC6439839

[24] E. Segelov , S. Thavaneswaran , P. M. Waring , et al., “Response to Cetuximab With or Without Irinotecan in Patients With Refractory Metastatic Colorectal Cancer Harboring the KRAS G13D Mutation: Australasian Gastro‐Intestinal Trials Group ICECREAM Study,” Journal of Clinical Oncology 34, no. 19 (2016): 2258–2264, 10.1200/JCO.2015.65.6843. 27114605

[25] M. Schirripa , F. Loupakis , S. Lonardi , et al., “Phase II Study of Single‐Agent Cetuximab in KRAS G13D Mutant Metastatic Colorectal Cancer,” Annals of Oncology 26, no. 12 (2015): 2503. 26371285 10.1093/annonc/mdv385

[26] E. Van Cutsem , C.‐H. Köhne , E. Hitre , et al., “Cetuximab and Chemotherapy as Initial Treatment for Metastatic Colorectal Cancer,” New England Journal of Medicine 360, no. 14 (2009): 1408–1417. 19339720 10.1056/NEJMoa0805019

[27] C. S. Karapetis , S. Khambata‐Ford , D. J. Jonker , et al., “K‐Ras Mutations and Benefit From Cetuximab in Advanced Colorectal Cancer,” New England Journal of Medicine 359, no. 17 (2008): 1757–1765. 18946061 10.1056/NEJMoa0804385

[28] B. O. Van Emburgh , A. Sartore‐Bianchi , F. Di Nicolantonio , S. Siena , and A. Bardelli , “Acquired Resistance to EGFR‐Targeted Therapies in Colorectal Cancer,” Molecular Oncology 8, no. 6 (2014): 1084–1094. 24913799 10.1016/j.molonc.2014.05.003 PMC5528615

[29] C. P. Vaughn , S. D. Zobell , L. V. Furtado , C. L. Baker , and W. S. Samowitz , “Frequency of KRAS, BRAF, and NRAS Mutations in Colorectal Cancer,” Genes, Chromosomes & Cancer 50, no. 5 (2011): 307–312. 21305640 10.1002/gcc.20854

[30] S. Edkins , S. O'Meara , A. Parker , et al., “Recurrent KRAS Codon 146 Mutations in Human Colorectal Cancer,” Cancer Biology & Therapy 5, no. 8 (2006): 928–932. 16969076 10.4161/cbt.5.8.3251 PMC2714972

[31] M. Janakiraman , E. Vakiani , Z. Zeng , et al., “Genomic and Biological Characterization of Exon 4 KRAS Mutations in Human Cancer,” Cancer Research 70, no. 14 (2010): 5901–5911. 20570890 10.1158/0008-5472.CAN-10-0192 PMC2943514

[32] G. Smith , R. Bounds , H. Wolf , R. J. C. Steele , F. A. Carey , and C. R. Wolf , “Activating K‐Ras Mutations Outwith “Hotspot” Codons in Sporadic Colorectal Tumours—Implications for Personalised Cancer Medicine,” British Journal of Cancer 102, no. 4 (2010): 693–703. 20147967 10.1038/sj.bjc.6605534 PMC2837563

[33] J. C. Jones , L. A. Renfro , H. O. Al‐Shamsi , et al., “Non‐V600 BRAF Mutations Define a Clinically Distinct Molecular Subtype of Metastatic Colorectal Cancer,” Journal of Clinical Oncology 35, no. 23 (2017): 2624–2630. 28486044 10.1200/JCO.2016.71.4394 PMC5549454

[34] S. D. Richman , P. Chambers , M. T. Seymour , et al., “Intra‐Tumoral Heterogeneity of KRAS and BRAF Mutation Status in Patients With Advanced Colorectal Cancer (aCRC) and Cost‐Effectiveness of Multiple Sample Testing,” Analytical Cellular Pathology (Amsterdam) 34, no. 1–2 (2011): 61–66. 21483104 10.3233/ACP-2011-0005 PMC4605581

[35] C. Santos , D. Azuara , R. Garcia‐Carbonero , et al., “Optimization of RAS/BRAF Mutational Analysis Confirms Improvement in Patient Selection for Clinical Benefit to Anti‐EGFR Treatment in Metastatic Colorectal Cancer,” Molecular Cancer Therapeutics 16, no. 9 (2017): 1999–2007. 28626084 10.1158/1535-7163.MCT-17-0153

[36] T. Sato , H. Osumi , E. Shinozaki , et al., “Clinical Impact of Primary Tumor Location and RAS, BRAF V600E, and PIK3CA Mutations on Epidermal Growth Factor Receptor Inhibitor Efficacy as Third‐Line Chemotherapy for Metastatic Colorectal Cancer,” Anticancer Research 41, no. 8 (2021): 3905–3915. 34281853 10.21873/anticanres.15186

[37] D. Tural , S. Batur , S. Erdamar , et al., “Analysis of PTEN, BRAF and PI3K Status for Determination of Benefit From Cetuximab Therapy in Metastatic Colorectal Cancer Patients Refractory to Chemotherapy With Wild‐Type KRAS,” Tumour Biology 35, no. 2 (2014): 1041–1049. 23996432 10.1007/s13277-013-1138-8

[38] S. E. Baldus , K.‐L. Schaefer , R. Engers , D. Hartleb , N. H. Stoecklein , and H. E. Gabbert , “Prevalence and Heterogeneity of KRAS, BRAF, and PIK3CA Mutations in Primary Colorectal Adenocarcinomas and Their Corresponding Metastases,” Clinical Cancer Research 16, no. 3 (2010): 790–799. 20103678 10.1158/1078-0432.CCR-09-2446

[39] L. Losi , B. Baisse , H. Bouzourene , and J. Benhattar , “Evolution of Intratumoral Genetic Heterogeneity During Colorectal Cancer Progression,” Carcinogenesis 26, no. 5 (2005): 916–922. 15731168 10.1093/carcin/bgi044

[40] V. Kosmidou , E. Oikonomou , M. Vlassi , et al., “Tumor Heterogeneity Revealed by KRAS, BRAF, and PIK3CA Pyrosequencing: KRAS and PIK3CA Intratumor Mutation Profile Differences and Their Therapeutic Implications,” Human Mutation 35, no. 3 (2014): 329–340. 24352906 10.1002/humu.22496

[41] A. R. Brannon , E. Vakiani , B. E. Sylvester , et al., “Comparative Sequencing Analysis Reveals High Genomic Concordance Between Matched Primary and Metastatic Colorectal Cancer Lesions,” Genome Biology 15, no. 8 (2014): 454. 25164765 10.1186/s13059-014-0454-7 PMC4189196

[42] D. Santini , F. Loupakis , B. Vincenzi , et al., “High Concordance of KRAS Status Between Primary Colorectal Tumors and Related Metastatic Sites: Implications for Clinical Practice,” Oncologist 13, no. 12 (2008): 1270–1275. 19056857 10.1634/theoncologist.2008-0181

[43] W. De Roock , B. Claes , D. Bernasconi , et al., “Effects of KRAS, BRAF, NRAS, and PIK3CA Mutations on the Efficacy of Cetuximab Plus Chemotherapy in Chemotherapy‐Refractory Metastatic Colorectal Cancer: A Retrospective Consortium Analysis,” Lancet Oncology 11, no. 8 (2010): 753–762. 20619739 10.1016/S1470-2045(10)70130-3

[44] A. Sartore‐Bianchi , F. Di Nicolantonio , M. Nichelatti , et al., “Multi‐Determinants Analysis of Molecular Alterations for Predicting Clinical Benefit to EGFR‐Targeted Monoclonal Antibodies in Colorectal Cancer,” PLoS One 4, no. 10 (2009): e7287. 19806185 10.1371/journal.pone.0007287 PMC2750753

[45] Cancer Genome Atlas Network , “Comprehensive Molecular Characterization of Human Colon and Rectal Cancer,” Nature 487, no. 7407 (2012): 330–337, 10.1038/nature11252. 22810696 PMC3401966

[46] F. Perrone , A. Lampis , M. Orsenigo , et al., “PI3KCA/PTEN Deregulation Contributes to Impaired Responses to Cetuximab in Metastatic Colorectal Cancer Patients,” Annals of Oncology 20, no. 1 (2009): 84–90. 18669866 10.1093/annonc/mdn541

[47] C. S. Karapetis , D. Jonker , M. Daneshmand , et al., “PIK3CA, BRAF, and PTEN Status and Benefit From Cetuximab in the Treatment of Advanced Colorectal Cancer—Results From NCIC CTG/AGITG CO.17,” Clinical Cancer Research 20, no. 3 (2014): 744–753, 10.1158/1078-0432.CCR-13-0606. 24218517

[48] G. Martini , D. Ciardiello , P. P. Vitiello , et al., “Resistance to Anti‐Epidermal Growth Factor Receptor in Metastatic Colorectal Cancer: What Does Still Need to Be Addressed?,” Cancer Treatment Reviews 86 (2020): 102023. 32474402 10.1016/j.ctrv.2020.102023

[49] S. Waldstein , M. Spengler , I. V. Pinchuk , and N. S. Yee , “Impact of Colorectal Cancer Sidedness and Location on Therapy and Clinical Outcomes: Role of Blood‐Based Biopsy for Personalized Treatment,” Journal of Personalized Medicine 13, no. 7 (2023): 1114. 37511727 10.3390/jpm13071114 PMC10381730

[50] M. E. Salem , F. Battaglin , R. M. Goldberg , et al., “Molecular Analyses of Left‐ and Right‐Sided Tumors in Adolescents and Young Adults With Colorectal Cancer,” Oncologist 25, no. 5 (2020): 404–413. 31848314 10.1634/theoncologist.2019-0552 PMC7216442

[51] D. L. Abravanel , G. K. Belka , T. Pan , et al., “Notch Promotes Recurrence of Dormant Tumor Cells Following HER2/Neu‐Targeted Therapy,” Journal of Clinical Investigation 125, no. 6 (2015): 2484–2496. 25961456 10.1172/JCI74883 PMC4497740

[52] A. Tyagi , A. K. Sharma , and C. Damodaran , “A Review on Notch Signaling and Colorectal Cancer,” Cells 9, no. 6 (2020): 1549. 32630477 10.3390/cells9061549 PMC7349609

[53] D. K. Dang and B. H. Park , “Circulating Tumor DNA: Current Challenges for Clinical Utility,” Journal of Clinical Investigation 15, no. 12 (2022): e154941. 10.1172/JCI154941 PMC9197509 35703177

[54] H. Kim and K. U. Park , “Clinical Circulating Tumor DNA Testing for Precision Oncology,” Cancer Research and Treatment 55, no. 2 (2023): 351–366. 36915242 10.4143/crt.2022.1026 PMC10101787

[55] A. Ottaiano , L. Circelli , and M. Caraglia , “The Tumor Dynamism Is the Dark Matter of the NGS Galaxy: How to Understand It?,” Cancers (Basel) 13, no. 21 (2021): 5476. 34771638 10.3390/cancers13215476 PMC8582436

[56] A. Ottaiano , M. Ianniello , M. Santorsola , et al., “From Chaos to Opportunity: Decoding Cancer Heterogeneity for Enhanced Treatment Strategies,” Biology (Basel) 12, no. 9 (2023): 1183, 10.3390/biology12091183. 37759584 PMC10525472

