# Supplementary material for: Search for Mutations Connected With Non‐Response to Anti‐EGFR Therapy in mCRC in the Morphologically Defined Regions of Primary Tumours
Source: Cancer Med. 2025 Apr 29;14(9):e70910. doi: 10.1002/cam4.70910 (PMC12040724; doi:10.1002/cam4.70910)
Supplement: Supplementary file 2 — File S1. [file CAM4-14-e70910-s002.docx]

**VCF files and clinical sample table:**

https://doi.org/10.5281/zenodo.10589454

**Demultiplexed fastq files (divided into 7 batches):**

https://doi.org/10.5281/zenodo.10589515

https://doi.org/10.5281/zenodo.10589646

https://doi.org/10.5281/zenodo.10590038

https://doi.org/10.5281/zenodo.10590151

https://doi.org/10.5281/zenodo.10590400

https://doi.org/10.5281/zenodo.10590592

https://doi.org/10.5281/zenodo.10590697
