# Supplementary material for: Search for Mutations Connected With Non‐Response to Anti‐EGFR Therapy in mCRC in the Morphologically Defined Regions of Primary Tumours
Source: Cancer Med. 2025 Apr 29;14(9):e70910. doi: 10.1002/cam4.70910 (PMC12040724; doi:10.1002/cam4.70910)
Supplement: Supplementary file 1 — Figure S1. Distribution of pathogenic variants in whole tumour, invasion front and across the morphological regions. Each row represents sample, each column represent mutations. Higlighted (bold) are mutations associated with non‐response to anti‐EGFR therapy. [file CAM4-14-e70910-s003.pdf]

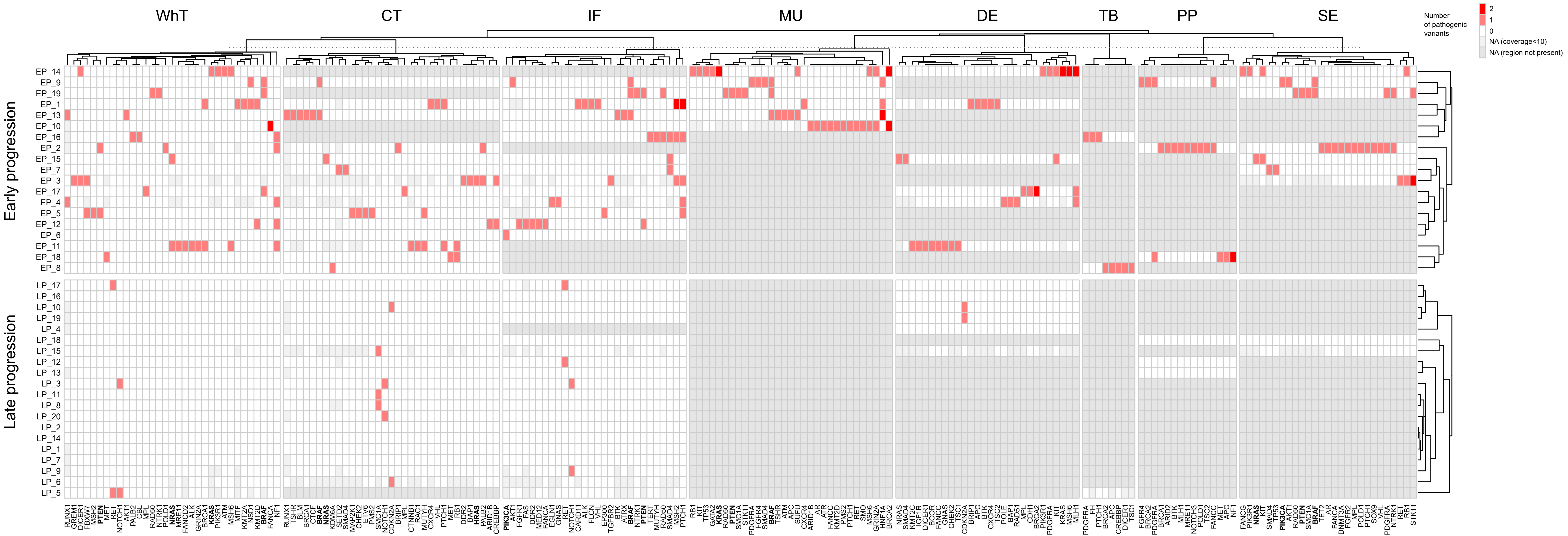

Figure S1. Distribution of pathogenic variants in whole tumour, invasion front and across the morphological regions. Each row represents sample, each column represent mutations. Higlighted (bold) are mutations associated with non-response to anti-EGFR therapy.
